# CT影像组学在肺癌诊治中应用的研究进展和问题探索

**DOI:** 10.3779/j.issn.1009-3419.2020.101.36

**Published:** 2020-10-20

**Authors:** 嘉威 李, 夏东 李, 雪琴 陈, 胜林 马

**Affiliations:** 1 310053 杭州，浙江中医药大学第四临床医学院肿瘤学专业 Zhejiang Chinese Medical University, Hangzhou 310053, China; 2 310002 杭州，杭州市肿瘤医院放疗科 Department of Radiation Oncology, Hangzhou Cancer Hospital, Hangzhou 310002, China; 3 310006 杭州，杭州市第一人民医院肿瘤科 Department of Oncology, Hangzhou First People's Hospital, Hangzhou 310006, China

**Keywords:** 肺肿瘤, 影像组学, 电子计算机断层扫描, 生物标记物, 预后, Lung neoplasms, Radiomics, Computed Tomography, Biomarkers, Prognosis

## Abstract

影像组学是一种基于多模态医学影像处理分析的技术，该技术能够基于高性能计算机及算法从目前普遍使用的计算机断层扫描（computed tomography, CT）、磁共振图像（magnetic resonance imaging, MRI）和正电子发射/断层图像（positron emission tomography/computed tomography, PET/CT）中自动提取海量数据进行分析，对疾病的早期诊断、良恶性肿瘤鉴别、疾病治疗全程管理，个体化精准治疗等需求提供更多有价值信息。近年来，许多研究表明基于CT的影像组学技术在肺癌的早期诊断、基因表型预测、疗效预测及预后评估均有良好的应用价值，且影像学检查具有无创、经济、可重复等优势。其对临床的指导价值已有所展露，在肺癌的个体化、精准化治疗和研究方面具有较大价值，但是，影像组学特征的重复性和一致性问题以及在肺部肿瘤图像提取中的特征筛选还需进一步研究。

## Abstract (en)

Radiomics, a technology based on multimodal medical image processing and analysis, is able to extract automatically and analyze massive data from computed tomography (CT), magnetic resonance imaging (MRI), positron emission tomography/computed tomography (PET/CT) via high-performance computer algorithm in order to pursue early diagnosis of disease, benign and malignant tumor discrimination, dynamic evaluation of disease treatment, and individualized precision therapy. To date, many studies demonstrate that radiomics not only has great potential in early diagnosis of lung cancer and prediction of genotype, treatment efficacy, as well as prognosis but also is based on imaging methods that are noninvasive, inexpensive, and repeatable. It does demonstrate precious values in guiding the clinical diagnosis and treatment of lung cancer, especially in the personalized and precise treatments and researches of lung cancer. However, the consistency and reproducibility of radiomics and the selection of robust characteristics still warrant further researches.

原发性肺癌是世界范围内最常见的恶性肿瘤，2018年全球共计约180万人死于肺癌，其中我国死亡人数约为69万，占死亡总人数约39%^[1]^。即使在发达国家肺癌患者的5年总生存率仍只有15%-18%^[2]^，有研究^[3]^称2020年美国预计将有超过13万人死于肺癌，居高不下的发病率及死亡率亟待有效的诊疗措施加以控制。影像组学是一门新兴学科，指通过高通量提取影像图像蕴含的参数结合机器学习算法提取影像学特征，筛选出临床价值最高的特征作为影像学特征用于解析肿瘤生物学特征以运用于临床，在肿瘤早期诊断、疗效评估中都开始有应用^[4, 5]^。影像组学的研究过程可分为以下四个步骤：图像的采集与重建，靶区域的勾画，特征的提取与筛选，建立预测预后的模型及验证^[6, 7]^。本文主要就影像组学在肺癌诊治过程中的应用研究进展及关键技术问题作一综述。

## 1 影像组学与良恶性肺结节鉴别

随着影像学检查的普及与技术提高，越来越多的肺部结节被检出，但鉴别精度有待提升，许多研究表明影像组学可以较好地鉴别良恶性结节。Mehdi等^[8]^想通过影像组学技术提取组学特征从而鉴别肺腺癌结节与肉芽肿结节，他们定义了一种新型组学特征名为定量血管迂曲（quantitative vessel tortuosity, QVT），该组特征能有效评估结节血管扭曲、丰富程度从而判定结节良恶性[训练集曲线下面积（area under the curve, AUC）=0.94±0.02；验证集AUC为0.85]。Hong等^[9]^通过研究发现，结合肺窗及纵隔窗的CT影像组学分析在鉴别惰性与侵袭性肺癌中更具优势。Yang等^[10]^研究发现CT影像组学特征结合临床指标可有效区分肺肉芽肿性结节与肺腺癌，该研究收集了302例患者，其中93例肉芽肿性结节，209例肺腺癌结节，按7:3的比例分为训练集与验证集。运用3D U-net模型进行结节分割，通过Pyradiomics软件共提取了94个影像学特征，为提高模型预测效能还加入年龄、性别、癌胚抗原（carcinoembryonic antigen, CEA）、糖类抗原125（carbohydrate antigen 125, CA125）、糖类抗原153（carbohydrate antigen 153, CA153）、结节大小等临床特征，分为6种模型：平扫组（plain radiomics, PR）、增强组（vein radiomics, VR）、平扫+增强组（plain radiomics+vein radiomics, PVR）、平扫+临床特征组（plain radiomics+clinical risk factors, PRC）、增强+临床特征组（vein radiomics+clinical risk factors, VRC）、平扫+增强+临床特征组（plain radiomics+vein radiomics+clinical risk factors, PVRC）。结果显示PRC组鉴别效能最高，训练集AUC为0.935，诊断敏感度为84.2%；验证集AUC为0.815，诊断敏感度为82.5%。上述研究提示影像组学特征虽然不能取代病理诊断，但对于一些形态不规则、呈分叶状、可疑恶性的结节可协助诊断指导临床治疗。

## 2 影像组学与肺癌生物标记物

靶向治疗在非小细胞肺癌（non-small cell lung cancer, NSCLC）治疗中占据着重要地位，基因检测是靶向治疗的前提，目前有研究正在探索利用影像组学进行基因分析的可能性。Yang等^[11]^研究发现CT影像组学特征是判断亚实性肺腺癌结节是否存在表皮生长因子受体（epidermal growth factor receptor, *EGFR*）突变的潜在有效指标。该研究纳入了467例经手术切除病理证实为肺腺癌的患者，分为训练集（306例）与验证集（161例），分析影像组学特征与*EGFR*突变的相关性，对特征进行降维后共提取了43个影像学特征，运用随机森林法建模，结果显示训练集及验证集AUC值分别为0.831与0.789，提示该模型与*EGFR*突变具有较好的相关性。Song等^[12]^通过分析335名NSCLC患者治疗前的计算机断层扫描（computed tomography, CT）图像提取影像特征以评估间变性淋巴瘤激酶（anaplastic lymphoma kinase, *ALK*）突变状态，发现结合影像组学特征、临床特征及传统CT特征建立的模型能够有效预测*ALK*突变状态（AUC为0.88）。Wang等^[13]^报道CT影像学特征作为提示早期肺腺癌患者肿瘤突变负荷（tumor mutation burden, TMB）与驱动基因突变的预测指标。该研究纳入51例已手术切除明确病理的患者（共计61个肺结节标本），运用二代测序（next generation sequencing, NGS）技术对标本进行*TMB*、*EGFR*及*TP53*突变检测，将TMB > 4定义为高^[14]^。为了进一步比较与优化模型的预测能力，研究人员还加入了临床数据组，包含年龄、性别、病理分期等临床特征，分为影像特征组、临床数据组及混合组。结果显示结合临床特征及影像特征的混合组具有较好的预测能力，TMB、EGFR、TP53的AUC值分别为0.671、0.697和0.656。虽然该模型由于样本量不足导致预测能力较低（AUC < 0.7），但之前的研究均没有对TMB的预测，也为以后的研究提供了指导。

免疫治疗也是肺癌的主要治疗手段，虽然免疫治疗展现出了良好的可耐受性并且能显著延长部分患者的生存期，但仍缺乏有效的生物标记物预测免疫治疗的应答率。而以程序性死亡因子-1（programmed death factor-1, PD-1）/程序性死亡因子配体-1（programmed death factor ligand-1, PD-L1）为靶点的免疫检查点抑制剂（immune checkpoint inhibitors, ICIs）主要通过检测PD-L1表达预测ICIs的疗效。Yoon等^[15]^探索是否能通过影像组学技术预测影像学特征与PD-L1表达的相关性，结果提示纹理特征（GLCM_ASM, GLRLM_RV, GLRLM_RE, GLRLM_SRHGE）结合临床特征（性别、年龄、吸烟史、*EGFR*突变状态）能较好的预测PD-L1的表达情况（c-statistic=0.646）。该研究同样存在选择偏倚、属于单中心研究、样本量不足等问题，如何挑选稳定的组学特征建模是未来的研究方向。

## 3 影像组学与肺癌疗效

### 3.1 预测肺癌放化疗

在评价传统治疗如放疗、化疗疗效方面，影像组学技术也有较好的应用价值。Lafata等^[16]^通过实验探索了影像组学特征预测NSCLC患者立体定向放射治疗（stereotactic body radio therapy, SBRT）疗效，结果显示同质性2（Homogeneity 2）（*P*=0.022）与长期高灰度级重点（Long-Run-High-Gray-Level-Emphasis）（*P*=0.048）与局部复发相关，多元*logistic*回归模型提示复发、局部复发、非局部复发的AUC值分别为0.72±0.04、0.83±0.03和0.60±0.04，说明该影像学特征更能预测是否发生局部复发。Elizabeth等^[17]^也进行了相似的研究，结果发现了3种传统特征和4种影像学特征是NSCLC患者SBRT治疗后的总生存率的预后因素，并且影像学特征还能预测远处转移的可能（CI=0.67, *q* < 0.1），这是传统图像参数无法预测的。对于小细胞肺癌（small cell lung cancer, SCLC），系统化疗仍然是主要治疗手段，虽然SCLC对化疗敏感且初始反应率≥60%，但5年生存率仍 < 5%。而且并非所有SCLC患者均对化疗有反应。Wei等^[18]^探索应用CT影像学分析预测SCLC患者接受一线化疗的应答率。结果显示影像组学模型（AUC为0.797）明显优于临床因素模型（AUC为0.670），证明影像组学特征可助临床医生更好的评估患者及选择更优的治疗手段。

### 3.2 预测肺癌靶向治疗

靶向治疗较传统放化疗具有较高安全性、方便及有效性的特点，*EGFR*基因突变患者采用酪氨酸激酶抑制剂（tyrosine kinase inhibitor, TKI）治疗有效率可达60%-80%，无进展生存期（progression-free survival, PFS）为8个月-12个月，然而并非存在*EGFR*突变的NSCLC患者均对靶向治疗有较好的反应。Song等^[19]^提出可以通过CT图像建立影像组学模型来预测Ⅳ期有*EGFR*突变的NSCLC患者使用EGFR-TKI治疗的PFS。他们发现了12种CT特征能够将患者区分为TKI治疗后快速进展组及TKI治疗后缓慢进展组，训练集、验证集1与验证集2的HR分别为3.61、3.77和3.67；此外快速进展组与化疗组相比在PFS方面无明显获益，可能为靶向联合化疗提供依据。

### 3.3 预测肺癌免疫治疗

免疫治疗目前是部分驱动基因阴性患者的标准治疗选择，其机制为通过激活人体自身免疫活性进行抗肿瘤应答，产生免疫记忆并有持续抗肿瘤作用，占据肺癌治疗的重要地位。然而NSCLC患者对免疫检查点抑制剂的反应率仍偏低，Trebeschi等^[20]^通过影像组学技术提取组学特征从而预测NSCLC患者使用PD-1抑制剂治疗的反应率。结果显示影像组学模型能较好预测对免疫治疗的反应率（AUC为0.76, *P* < 0.001）。此外，Tunali等^[21]^通过CT影像组学技术建立的模型能够预测NSCLC患者接受免疫治疗后出现超进展的风险，该研究纳入了228例使用PD-1或PD-L1或联用CTLA-4的NSCLC患者，综合分析了临床特征及影像学特征，发现接受免疫治疗之后是否出现超进展与肝转移、骨转移、过往治疗方案、中性粒细胞与淋巴细胞比率、径向梯度边界SD-2D（radial gradient border SD-2D）、三维Laws纹理学特征E5L5E5（3D Laws E5L5E5）、三维边界Laws纹理学特征E5E5L5（border 3D Laws E5E5L5）以及边界四分位离散系数（border quartile coefficient of dispersion）有关（AUC为0.865，特异度为92.9%，敏感度为74.0%）。

免疫相关性不良反应是目前肿瘤科医生最关注的不良反应，3级以上免疫相关性肺炎虽然发生率较低，但可危及生命。Colen等^[22]^通过研究证实了可以使用CT影像组学特征来预测免疫相关性肺炎发生的风险，该研究共纳入了32例使用免疫疗法的实体瘤患者（包含2例免疫性肺炎患者），分析两组患者CT影像学特征有无差异，结果发现偏度（skewness）与平方和角方差（angular variance of sum of squares）两种特征与免疫性肺炎相关性最高（AUC为1，*P*=0.003, 3），认为影像组学分析免疫性肺炎的发生具有潜在的预测价值。

## 4 影像组学与肺癌预后

肺癌的预后与许多因素相关，如个体因素、肿瘤原发灶-淋巴结-转移（tumor-node-metastasis, TNM）分期、病理结果、治疗手段、是否转移等，但大多属于定性因素，而影像组学运用计算机算法更有利于将肺癌患者的预后情况可视化。已有相关研究通过影像组学技术提取的特征来描述肿瘤表型并且直接预测肺癌患者的预后。Wang等^[23]^研究发现逆差矩（GLCM-inverse different moment）、ASM能量（GLCM-angular second moment）及分叶征等特征与肿瘤异质性及侵袭性相关，能较好预测肺癌患者的生存期。此外，Evelyn等^[24]^研究发现使用Ⅰ期-Ⅲ期NSCLC患者的CT影像数据建立的影像组学特征，对单纯化疗的Ⅳ期腺癌患者同样有总生存期（overall survival, OS）预测作用（HR=1.46, 95%CI: 1.02-2.10, *P*=0.04）。晚期肺癌患者较常发生远处转移，通常有远处转移提示预后不佳，目前有不少研究者尝试通过影像组学分析预测肿瘤转移。Chen等^[25]^将平扫CT图像进行组学分析，发现组学特征能够预测NSCLC患者发生脑转移（brain metastasis, BM）的风险。He等^[26]^称通过影像组学特征建立的危险预测评分系统（radiomics-based predictive risk score, RPRS）可有效评估可手术切除的NSCLC患者术后出现淋巴结转移的可能性，为下一步治疗提供指导。Coroller等^[27]^报导称可通过CT影像组学特征预测肺腺癌患者远处转移的风险。他们共纳入182例经病理确诊为肺腺癌的患者，设置的主要研究终点为出现远处转移（distant metastasis, DM），次要研究终点是OS。该研究同样结合了一些可能与肿瘤远处转移相关临床变量，如肿瘤分级、美国东部肿瘤协作组（Eastern Cooperative Oncology Group, ECOG）、TNM分期等，结果显示在所有的特征中有35个特征与DM关系密切（CI > 0.60且*P* < 0.05），12个特征与OS相关。但是本研究也有一定的局限，如纳入的患者时间跨度较长，为2001年-2013年，期间CT机器及图像重建算法可能存在变化，得到的图像数据存在差异。但研究结果还是提示影像组学特征与DM有密切关系，为以后患者预测预后、选择合适治疗方案提供指导。

## 5 结语与展望

影像组学技术在肺癌的诊治方面展现了不错的应用前景，但仍缺乏多中心前瞻性影像组学研究以指导临床实践，其原因就在于影像组学研究方法缺乏“金标准”，影像组学特征的可重复性得不到认可。研究表明图像重建算法、预处理方式、传输协议、观察者间变数、特征提取算法等均可影响影像组学特征的稳定性及可重复性。如何解决可重复性问题是未来研究的关键，根据影像组学研究过程可分为以下四个方面：①图像获取及重建的可重复性；②靶区域勾画的可重复性；③特征提取算法的可重复性；④研究的可重复性。在进行影像组学研究时第一步即获取及重建图像，不同的扫描机器、扫描参数、呼吸运动都可能造成干扰，对此可通过统一扫描策略、应用4DCT技术以获得标准的影像图像从而提高所提取的影像特征的稳定性。对于靶区勾画，已有研究证实通过半自动勾画提取的影像组学特征可重复性明显优于手动勾画，然而最理想的勾画方式仍然是全自动勾画，其不仅能降低工作量，更能消除观察者间差异，提高特征可重复性^[28]^。目前基于深度学习的自动勾画技术已取得了长足进步，并逐渐应用于医学图像分析，但仍需解决如何剔除影响勾画精度的影像学特征的问题^[29]^。特征提取主要分为预处理及特征计算两步，已有部分研究^[30, 31]^提出不同预处理方法将对特征提取产生影响，为此有研究团队提出了生物标志物标准化倡议（Image Biomarker Standardization Initiative, IBSI）以标定特征提取及预处理模式基准，消除上述差异带来的研究误差^[32]^。最后是研究的可重复性，需要建立一个数据公开化、研究透明化的开源软件平台，目前已有Pyradimics、Insight ToolKit、Imaging biomarker Explorer等平台在不断完善以实现影像组学研究的可重复性。

影像组学仍处于不断发展的阶段，但其展现的巨大价值已不容忽视，若能克服上述问题，肺癌的早期诊断与个体化精准化治疗将真正得以体现。
